# Representing the UK's cattle herd as static and dynamic networks

**DOI:** 10.1098/rspb.2008.1009

**Published:** 2008-10-14

**Authors:** Matthew C. Vernon, Matt J. Keeling

**Affiliations:** Department of Biological Sciences, University of WarwickCoventry CV4 7AL, UK

**Keywords:** dynamic networks, transmission, infection, livestock movements

## Abstract

Network models are increasingly being used to understand the spread of diseases through sparsely connected populations, with particular interest in the impact of animal movements upon the dynamics of infectious diseases. Detailed data collected by the UK government on the movement of cattle may be represented as a network, where animal holdings are nodes, and an edge is drawn between nodes where a movement of animals has occurred. These network representations may vary from a simple static representation, to a more complex, fully dynamic one where daily movements are explicitly captured. Using stochastic disease simulations, a wide range of network representations of the UK cattle herd are compared. We find that the simpler static network representations are often deficient when compared with a fully dynamic representation, and should therefore be used only with caution in epidemiological modelling. In particular, due to temporal structures within the dynamic network, static networks consistently fail to capture the predicted epidemic behaviour associated with dynamic networks even when parameterized to match early growth rates.

## 1. Introduction

The movement of animals within the UK is vital to the economics of the livestock industry, but carries with it the risk of transmitting infectious diseases across substantial geographical distances. Data on the movement of all cattle in the UK are collected by the Department for Environment Food and Rural Affairs (DEFRA), as part of the Rapid Analysis and Detection of Animal-related Risks (RADAR) system, itself part of the Veterinary Surveillance Strategy (Lysons *et al*. 2007).

This movement data may be abstracted into a directed contact network in which agricultural premises are nodes, and the movements of cattle between premises are edges. The resulting network may be analysed using a range of techniques, including those developed for handling social networks (Wasserman & Faust 1994; Carrington *et al*. 2005). A common approach has been to consider all the movements within a fixed period (typically 7 or 28 days, or a year) as a static network, and then to analyse the properties of the resulting network (Christley *et al*. 2005; Bigras-Poulin *et al*. 2006), or to repeat this process for a consecutive sequence of such periods and look for trends in the properties of the resulting networks (Robinson *et al*. 2007). Indeed, most social network analysis concentrates on static networks, and there is a paucity of strategies for addressing the structure of dynamic networks (Wasserman & Faust 1994). Research into dynamic networks has concentrated on models based on how individuals create or change their ties in a network, in response to their perception of that network's structure (Snijders 2005), how popular other individuals in the network are (Barabási & Albert 1999), their social distance from and shared activities with other individuals (Kossinets & Watts 2006) or how the other individuals perform in a game-theoretic framework (Skyrms & Pemantle 2000; Zimmermann *et al*. 2004). The dynamic pattern of movement between farms is also likely to be governed by some underlying set of rules linking livestock population dynamics with economics; however, given the comprehensive nature of the recorded movements, our aim is to understand how they influence disease transmission.

The UK cattle movement data, and the network of connections that can be derived from it, are one of the most detailed datasets available on dynamic network structure. As such, these data have provided an ideal test of many theories and concepts from network theory. What is more, the presence of information about infection on cattle farms (Wint *et al*. 2002; Gilbert *et al*. 2005) provides a real-world comparison with the ideals of network theory. Obviously predicting the spread of actual infections through the cattle movement network requires models that can accurately capture the epidemiology and natural history of a particular pathogen, and produce results that are specific to the particular infection studied. Here we adopted an alternative, and more generic approach, using simple disease models to understand the implications of dynamic cattle movements, as opposed to static network connections. These simple models treat the farm as a single epidemiological unit.

In this paper, a range of static and dynamic network representations of the UK's cattle herd are considered. Since the purpose of constructing network models of cattle movement is to understand the impact of movements upon the dynamics of infectious disease, simulated disease processes were employed to assess the suitability of the different network representations. More specifically, a stochastic, discrete-time susceptible, infectious, recovered (SIR) disease model was developed, and the dynamics and final epidemic size of simulations run upon the different network representations measured. Our aim was to ascertain whether any static network provides a consistent approximation to the fully dynamic network, or to identify regions of epidemiological parameter space where static network approximations may be valid.

## 2. Material and methods

### (a) Movement data

Cattle movement data were provided by DEFRA from the RADAR project on 24 May 2006. In this analysis, only movements occurring during 2004 were considered.

The main information in this database is a ‘livestock location’ table, with each row containing the following information: the identity of the location and animal, the arrival and departure dates, the type of arrival and departure movements (including details of how they were inferred, if relevant) and the country imported from or exported to, if relevant. To derive movements (the edges in a contact network) from this table, it was necessary to find two stays on locations where the animal concerned is the same, and the end date of one stay is the start date of the other; additionally, the start and end locations of the movement should be different, and the movement type by which the animal arrives at the destination holding should not be birth or death.

In particular, we translated the movement of individual cattle between farms into a network of nodes and edges, and then translated the edges into a graph-theoretic matrix presentation, *G*_*i*,*j*_(*d*), which defines the strength of the connection from premise *i* to premise *j* on day *d* (generally *G*_*i*,*j*_(*d*) will be 1 if there was a movement of cattle from *i* to *j* on day *d* and 0 otherwise, although we use the term strength as *G*_*i*,*j*_ can take other values in alternative network representations). We note that the matrix is not symmetric as movements have a definite direction associated with them.

### (b) Disease simulation

The spread of disease on the network representations discussed in this paper was modelled using a simple stochastic discrete-time SIR model. Our model treated farms as a single unit, comparable with the basic assumptions within the models developed for the 2001 foot-and-mouth epidemic in the UK (Kao 2002; Keeling 2005). This is akin to a simple Levins-type metapopulation model (Levins 1969) in which each farm exists in one of three basic states. In addition, all farms were considered identical, such that neither number of cattle, breed nor farming practices have any effect on the transmission dynamics; this is obviously a crude assumption, but allows us to examine the impact of network structure in isolation from other heterogeneities. Most nodes began the simulation in the susceptible (S) state, although a small number, *α* (set to 1 throughout this paper), were chosen at random to begin in the infected (I) state. The model was then synchronously updated using a daily time step. During each time period, disease passed along each edge from an I node to an S node with probability *ν*. Nodes remained in state I for an integer number of iterations, *μ*, and then passed into the recovered (R) state. Nodes in the R state remained in that state forever. The parameters *ν* and *μ* remained constant during any given simulation. In the case of dynamic networks, the network was updated after every model *time* step.

Formally, the dynamics can be described as follows:

(2.1) $$\begin{array}{l} p(\text{state}(i,t+1)=\text{I}\;|\text{state}(i,t)=\text{S})=1\;-\prod_{\text{state}(j,t)=\text{I}}\left(1-\nu G_{j,i}\right)\\ p(\text{state}(i,t+1)=\text{R}\;|\text{state}(i,t-\mu )\neq \text{S})=1, \end{array}$$

where state(i,t)∈{S,I,R} is the state of node *i* at time *t*. As such, it is clear that only the infection process (first line of equation (2.1)) depends on the network structure, while recovery is independent, operating at the farm level.

This model is implemented using the functions sir_net and sir_dynamic_net (for static or dynamic networks, respectively) in the ‘Contagion’ software package (Vernon 2007).

### (c) Network representations

The cattle movement data from 2004 were abstracted to form networks in six different ways. In general, these networks either represented plausible approximations to the fully dynamic network or allowed the exploration of various aspects of the fully dynamic network. In each case, agricultural premises (such as farms or slaughterhouses, but not markets where stays are generally too short to result in transmission) were represented as nodes, and movements of cattle were represented as directed edges (edge direction being the same as the direction of cattle movement). For each resulting network, 10 000 disease simulations were run with values of *ν* ranging from 0.01 to 1 at intervals of 0.01 and with values of *μ* ranging from 1 to 50 (time steps, which are equal to days) at intervals of 1 (a total of 50 million simulations per network).

For each of the networks defined below, we determined a graph matrix representation (*G*) that was related to the recorded pattern of movements. We represented the recorded movements as

$${\hat{G}}_{ij}(d)=\{\begin{array}{ll} 1, & \text{if}\;\text{movement}\;\text{from}\;i\;\text{to}\;j\;\text{on}\;\text{day}\;d,\\ 0, & \text{otherwise}. \end{array}$$

Hence $\hat{G}(d)$ was an *N*×*N* matrix linking the *N* livestock premises in Great Britain. We note that $\hat{G}(d)$ is solely based on the presence or absence of movements on a given day and does not capture the number of animals that are moved.

#### (i) Dynamic

The dynamic network (*G*^full^) was used to represent the consequences of all 366 days' movements for 2004. In practice, the dynamic network was effectively 366 static networks, one for each day of the year; if cattle moved from farm *i* to farm *j* on day *d*, then the network for day *d* would contain an edge *i*→*j*. To accommodate long-duration epidemics that lasted more than 1 year, the dynamic network was made periodic. We therefore set

$$G^{\text{full}}=\langle \hat{G}(0),\ldots ,\hat{G}(365)\rangle ,$$

where 〈…〉 denotes an ordered set. We considered the behaviour predicted by the dynamic network to be our ‘gold standard’; while acknowledging that our epidemiological assumptions are too simplistic to match any real infection, the dynamic network most faithfully captures the true pattern of contacts between farms.

#### (ii) Periodic dynamic

This network representation was constructed in the same manner to the full dynamic networks, but only movements from a limited number of days (either 7 or 28) were considered. The periodic-dynamic network representation *G*^pd^(*x*_1_, *n*), for a period of *n* days starting on day *x*_1_ was defined as

$$G^{\text{pd}}(x_{1},n)=\langle \hat{G}(x_{1}),\ldots ,\hat{G}(x_{1}+(n-1))\rangle .$$

As such, comparing results from the periodic dynamic network with those from the full dynamic network allowed the assessment of the degree of variation in network structure throughout the year. The periodic dynamic network captured the full movement pattern from a short interval; the issue is whether such an interval is representative of a year. Generally, we take *x*_1_=0 and either *n*=7 or 28 days.

#### (iii) Static

This network was by far the simplest one considered. A number of days' movements (either 7 or 28) were combined, such that any movement of animals between two premises within that period would result in an edge between the nodes corresponding to those premises in the network. The static network representation *G*^stat^(*x*_1_, *n*), for a period of *n* days starting on day *x*_1_ was therefore defined as

$$G_{ij}^{\text{stat}}(x_{1},n)=\{\begin{array}{ll} 1, & \text{if}\;\sum_{d=x_{1}}^{x_{1}+(n-1)}{\hat{G}}_{i,j}(d)\geq 1,\\ 0, & \text{otherwise}. \end{array}$$

This static network did not take into account the number of times a dynamic connection was present and was therefore expected to substantially overestimate transmission compared with its fully dynamic counterpart for the same epidemiological parameter values.

#### (iv) Weighted static

The weighted static network represented a straightforward refinement of the previous static network, but accounted for the assumption that the frequency of movements between farms is likely to be relevant to disease transmission. It was constructed in the same manner as the static network representation, but the resulting edges were given a weight equal to their frequency in the time period considered. The weighted static network representation *G*^ws^(*x*_1_, *n*), for a period of *n* days starting on day *x*_1_ was again an *N*×*N* matrix, the entries of which were defined as

$$G_{ij}^{\text{ws}}(x_{1},n)=\frac{\sum_{d=x_{1}}^{x_{1}+(n-1)}{\hat{G}}_{i,j}(d)}{n}.$$

In addition to the standard *n*=7 and 28-day periods, a weighted static network was constructed considering all movements in 2004 (*n*=366). In many ways, the weighted static network represented the natural static version of the fully dynamic network (Bell *et al*. 1999; Corner *et al*. 2003). The key issue is the effect of replacing the brief strong connections of the dynamic network with permanent weaker connections in the static model. Although both network assumptions should lead to the same expected transmission from a given infected farm, the timings and distributions of secondary cases were anticipated to be very different.

The final two network representations examined ways in which the dynamic network could be smoothed. As such, they provided a simple test of the implications of daily movement structure as opposed to more slowly varying network structures.

#### (v) Sequential weighted static

This representation consisted of a series of weighted static networks, each being used for the number of simulation time steps equal to the number of days' movements it had been constructed from. For example, where 7-day weighted static networks were used, the first seven simulation time steps would be run on the weighted static network constructed from days 1–7 of the original movement data, the second seven simulation time steps on the weighted static network constructed from days 8–14 of the original movement data, and so on. For this representation, due to computational overheads, only 1000 simulations were performed for each *ν* and *μ* value. The sequential weighted static representation considering *n* days, *G*^sws^(*n*), was defined thus

$$G^{\text{sws}}(n)=\langle GW(0),\ldots ,GW(X)\rangle ,\;\text{where}\;GW(x_{1})=G^{\text{ws}}\left(n\left\lfloor \frac{x_{1}}{n}\right\rfloor ,n\right),$$

where ⌊*x*⌋ represents the integer value of *x*, rounding down.

#### (vi) Smoothed

The smoothed network consisted of a series of weighted static networks, one per day, to effectively produce a moving average of the fully dynamic network. For example, using a 7-day moving average, the first network in this representation was a weighted static network constructed from days 1–7 of the original movement data, the second was a weighted static network constructed from days 2–8 of the original movement data, and so on. Again, both 7- and 28-day moving averages were considered. For this representation of the network, only 1000 simulations were performed for each *ν* and *μ* value. The smoothed network representation using a moving average over *n* days, *G*^smooth^(*n*) was defined as

$$G^{\text{smooth}}(n)=\langle G^{\text{ws}}(0,n),G^{\text{ws}}(1,n),\ldots ,G^{\text{ws}}(365,n)\rangle .$$

## 3. Results

Our first observation concerns the difference between 7- and 28-day based networks. Throughout, for greater clarity of the figures, we only show results from 28-day networks. Smoothing using 7- and 28-day windows generated similar behaviours. Epidemics run upon the 7-day periodic-dynamic, static and weighted static representations behaved similarly to those run on the equivalent 28-day representations, but with a smaller final epidemic size (data not shown). This is to be expected as the shorter 7-day sampling interval leads to fewer movements being included and therefore a network which is not as well connected.

### (a) The effect of varying infectious period when transmission probability is constant

Figure 1*a*,*b* shows mean final epidemic size against infectious period for a transmission probability of 0.3 and 0.7, respectively; comparable results are obtained for all transmission probability values investigated. When transmission probability was relatively low (as in figure 1*a*), disease simulations upon the (28-day) static network representations resulted in significantly larger final epidemic sizes than those upon other network representations; this effect was especially marked with short infectious periods. The static network representation combined multiple days' movements into one single network, resulting in a comparatively dense network; accordingly a relatively large number of nodes were infected, even during a short-lived epidemic. For all but the smallest infectious periods, the static network gave rise to an approximately constant final epidemic size (of approx. 3000 farms); this signified that the epidemic had reached all available nodes within the network—in this case it was the sample size of 28 days and not the transmission process that limited the epidemic. This means that epidemics generated on networks that used all the movements in 2004 could potentially exceed 28-day static network epidemics if the infectious period and transmission probability were large enough.

Other networks based on 28-day samples (the periodic-dynamic and 28-day weighted static network representations) produced results that approached asymptotically to those of the static network as the infectious period became sufficiently long. However, for shorter infectious periods, both of these models produced smaller epidemic sizes due to the weaker strength of connections (in the case of the weighted static) or intermittency of connections (in the case of the periodic-dynamic network). Interestingly, the periodic-dynamic network consistently produced larger epidemics than the weighted static, due to the way that the fixed infectious period interacted with daily movements.

The two smoothed networks generated similar sized epidemics to the full dynamic network; with all three showing increasing final epidemic size with increasing transmission probability and infectious period.

For low transmission rates, the year-long weighted static network (the most natural static approximation) produced final epidemic sizes similar to those of the full dynamic model; hence it might be argued that, in terms of this simplest measure, the weighted static network performs well. However, as the transmission probability increased, the weighted static network produced far larger epidemic sizes. This discrepancy is due to which element limits the epidemic spread—when transmission rates are high, spread through the dynamic network was limited by the intermittent presence of connections, whereas for the year-long weighted static network, connections were always present and it was the probabilistic nature of transmission that limited the infection process. This argument is made more precisely later.

### (b) The effect of varying transmission probability when infectious period is constant

Figure 2 again shows final epidemic size, but now the infectious period is fixed (at *μ*=50 days) and the transmission probability is varied. A similar pattern is visible here as in figure 1*a*,*b*—however, it is more noticeable that both of the smoothed networks underestimated the final epidemic size predicted by the fully dynamic network. This underestimation was in part due to the way that transmission probabilities were modified by the smoothed networks. For the extreme case where the transmission probability *ν*=1, a single connection in the dynamic network was guaranteed to transmit infection (assuming the farm is infectious); however, this was not the case for the smoothed networks where the reduced transmission rate (over a longer period) meant that infection may fail to transmit.

### (c) Differences in epidemic time courses between different network representations

Turning to the epidemic dynamics in more detail, figure 3*a* illustrates typical time courses for outbreaks simulated on the various network representations. It shows the mean number of recovered nodes (total epidemic size so far) at each time step from simulations run with a transmission probability of *ν*=0.36 and an infectious period of *μ*=12 days; lines stop when the epidemic dies out. Figure 3*b* shows the same information, but with the static network representation result removed, for clarity. These figures give the clearest indication so far that the different networks give rise to different epidemic profiles; as expected, for these parameters the static network produced by far the largest and most rapid epidemic. In all cases, the epidemics followed the typical sigmoidal time course of an SIR epidemic—initial slow spread, followed by a period of rapid growth, which then slowed again as the susceptible population was depleted (Anderson & May 1991). It is interesting to note that the weekly farming cycle is observable in the dynamic network with far less transmission occurring on Sundays; a similar feature is seen for the periodic-dynamic network.

### (d) The differences between the network representations are not merely a matter of scaling

It is not clear from the above results whether epidemics on different network representations are systematically different, or merely represent different scalings of the underlying parameters. To address this question, we looked for a consistent pattern between early growth and final epidemic size across all networks. Figure 4*a* enables this question to be addressed, plotting final epidemic size against the number of infectious nodes after one infectious period (comparable with *R*_0_) across the full range of transmission probability and infectious period values (each point represents the outcome of a single model run). The relationship between early epidemic growth and final epidemic size was different for all the different representations (excepting the smoothed and sequential weighted static representations, which are similar to each other in this regard). In figure 4*b*, the *x*-axis is a log scale, which clarifies the differences between the year-long weighted static representation and the dynamic representation for smaller epidemic sizes. These figures highlight the fact that the differences between networks are not due to a simple rescaling of transmission probabilities, but a more subtle interplay between total probability of transmission, time to infection and the scale of the interconnected network.

### (e) Theoretical considerations

We now use some simple analytical calculations to interpret the differences observed so far, focusing in particular on the somewhat unexpected differences between dynamic and weighted static networks.

Traditionally, analytical techniques for considering disease spread through networks are based upon concepts from percolation theory—which itself assumes that the network is static and assigns probabilities to each link. However, to understand the differences between dynamic and static networks, we need to work from first principles in considering the spread of infection between nodes (farms). Consider the contacts and interaction between two farms; one of the simplest situations is whether animals are moved between them just once in a year. In the fully dynamic network, $G_{ij}^{\text{full}}(d)$ will be 1 on the day of movement and 0 on all 365 other days; by contrast, the year-long weighted static network will have $G_{ij}^{\text{WS}}=1/366$ for all time points. Comparing these two network representations we see that the probability of transmission is given by

$$p^{\text{full}}=\nu \frac{\mu }{366}\;\;p^{\text{WS}}=1-{\left(1-\frac{\nu }{366}\right)}^{\mu },$$

such that there is a nonlinear scaling between the two probabilities and *p*^full^≥*p*^WS^. (We note that the two probabilities are equal whenever *μ*=1). The ratio of these probabilities at the level of individual contacts can be translated into relative population-level epidemic sizes, with the clear prediction that higher transmission probabilities should (on average) lead to larger epidemic sizes—this is observed when comparing the 28-day periodic-dynamic with 28-day weighted static representations in figures 1–3.

The calculation of transmission probabilities can also be extended to the situation where there are *n* movements from one farm to the other; assuming that movements occur at random throughout the year we have that

$$p_{n}^{\text{full}}=\sum_{m=1}^{n}\left(\begin{array}{c} m\\ n \end{array}\right)\frac{(366-n)!\mu !(366-\mu )!}{366!(\mu -m)!(366+m-n-\mu )!}[1-{\left(1-\nu \right)}^{m}]\;\;p_{n}^{\text{WS}}=1-{\left(1-\frac{\nu n}{366}\right)}^{\mu }.$$

Although these forms are more complex, it can be shown that, as before, the fully dynamic model has a higher probability of transmission compared with the weighted static network and therefore it is expected to generate larger epidemics; this effect may be observed in the results from artificially created dynamic networks and their associated year-long weighted static equivalents. In addition, it can be readily seen that a weighted static network sampled over a shorter time scale has a lower transmission probability compared with the year-long version.

For the case when *n*=1, we can also calculate the time to infection (assuming infection has occurred)

$$T^{\text{full}}=\frac{\mu +1}{2}\;\;T^{\text{WS}}=\sum_{i=1}^{\mu }\text{i}{\left(1-\frac{\nu }{366}\right)}^{i-1}\frac{\nu }{366p^{\text{WS}}},$$

and hence we find that the weighted static network is likely to transmit infection more rapidly. When *ν* and *μ* are both large (and noting that we are assuming *n*=1), we observe that transmission is likely in both models but occurs far more rapidly in the weighted static model.

We now compare these theoretical results with our simulation studies. Two of our theoretical predictions are supported: (i) the year-long weighted static network gives rise to larger epidemic sizes than weighted static networks sampled over shorter time scales, (ii) the year-long weighted static network gives rise to epidemics that grow much more rapidly than the fully dynamic network (and faster than shorter weighted static networks). However, in contrast to our theoretical predictions, we find that the year-long weighted static network gives rise to larger epidemics than the fully dynamic network. Detailed analysis of the causes for this theoretical failure highlights the inaccuracy of our assumption (for the case where *n*>1) that movements occur randomly throughout the year; the true pattern of movements from a given farm shows both positive and negative correlations at a range of temporal lags. This temporal pattern reflects both livestock management (and dynamics) on the farm and legal constraints on the movement of livestock. In particular, the 6-day standstill period prevents multiple on- and off-movements within a 6-day period, while the natural cycle of births leads to increased number of movements in both spring and autumn. We therefore observe that the temporal correlation between movements to and from a farm leads to a significant reduction in disease spread compared with a random pattern of movements, which is the primary aim of the legal restrictions on animal movements (Madders 2006).

### (f) Distribution of epidemic sizes

One applied use of such between-farm movement networks is to examine the early spread of foot-and-mouth disease (Green *et al*. 2006). Foot-and-mouth disease is unlikely to go undetected for more than four weeks, and so weighted static networks for a 28-day period have been used to model the early spread of infection. Given the arguments above concerning the differences between static and dynamic networks, we would in general hope that using a shorter interval for both networks would lead to greater similarity—given that 1-day networks will be identical. It is therefore reasonable to consider the suitability of simpler network representations for modelling such truncated epidemics.

A rapid infectious disease was simulated, with parameters (*ν*=0.9, *μ*=8) chosen such that the final epidemic size between the 28-day weighted static representation and the dynamic representation was comparable. The simulated epidemics were halted after 28 days, and one hundred million disease simulations were run. Figure 5*a* shows the frequency distribution (on a log scale) of epidemic size after 28 days from these simulations. The mean final epidemic size for the dynamic network representation was 121, and for the 28-day weighted static representation was 155. A two-sample Kolmogorov–Smirnov test (Conover 1999) shows that these two distributions are significantly different (*p*<2.2×10^−16^). We can also conclude, simply from the differences between the means, that for the same parameter values, epidemics simulated through dynamic and weighted static networks do not agree even at the shorter 28-day time scale.

To generate a more fair comparison, the transmission probability within the 28-day weighted static network was changed to achieve agreement between the mean epidemic sizes predicted by the two network representations. One hundred million disease simulations were again run with this new transmission value (*ν*=0.8327) on the 28-day weighted static network representation, and the results plotted against the original dynamic network representation simulation outputs as figure 5*b*. Although the mean final epidemic size was 121 in both cases, a two-sample Kolmogorov–Smirnov test again showed that the two distributions were significantly different (*p*<2.2×10^−16^).

The differences between the weighted static and dynamic network representations in figure 5*a*,*b* are particularly noticeable at the higher final epidemic sizes, which would lead to the worst-case scenario being considerably underestimated if a weighted static network representation were used to inform policy making. The peaks observed in the dynamic network representation are an interesting example of the importance of the dynamic nature of cattle movement. If a single movement acts to connect two large interconnected groups of farms, then in a dynamic model transmission between the two groups relies on infection reaching the interconnecting link at the appropriate time. Those epidemics that reach the link at the appropriate moment and therefore infect both groups of farms are likely to give rise to far larger epidemics than those that fail to reach the link—leading to bimodal distributions of epidemic sizes. This sort of dynamic effect is lost in static network representations, yet may be important to understand the dynamics of infectious diseases in the UK cattle herd. With hindsight, this bimodal nature is observable in figure 4*b* for the dynamic network.

## 4. Discussion

The cattle movement network from the UK provides one of the most detailed examples of a well-documented network that has been continuously sampled over an extended period. As such, it provides an ideal test of many ideas about dynamic networks, and how they can be understood and analysed. In particular, there are clear resonances with human contact networks, where connections are often seen as static, but in practice contacts only occur intermittently. The key question is whether this complex dynamic pattern of interactions can be captured by a suitable scaling of a static network or whether the dynamic complexities have to be modelled explicitly for their effects to be captured.

Figures 1 and 2 show that the different network representations of the UK cattle herd exhibit differing behaviours as the two simulation parameters (infection probability and infectious period) are varied. Therefore, for a given set of epidemiological parameters, which set the local dynamics, no other representation was able to capture the population-level behaviour. Moreover, by plotting early epidemic growth against final epidemic size (figure 4*a*), we have shown that these differences are systematic and cannot be removed by a simple rescaling of epidemiological parameters; even if network models are all parameterized to match the same observed early epidemic behaviour, they fail to agree with predictions of final epidemic size. This shows that the differences between the epidemics reflect fundamental differences in the way that the infection dynamics interact with the network properties.

Finally, we compared weighted static network models with results from the dynamic network and consider a scenario designed to minimize the differences. Both network models are simulated for just 28 days (minimizing the impact of longer term temporal correlations) and the epidemiological parameters are determined such that the mean epidemic size (at the end of 28 days) is in agreement. However, despite these measures, we still observe significant differences between the distributions of epidemic sizes, with the dynamic network predicting more extreme values.

While simpler network representations of the UK cattle herd have their advantages, these results show that great care must be taken if such representations are to be used for epidemiological prediction. We have considered a range of alternatives to the most realistic representation (i.e. the fully dynamic network), and shown that they are defective even when considering a relatively simple SIR disease simulation. In particular, when comparing fully dynamic network models to their weighted static equivalent (probably the most natural approximation), we find that the temporal correlations between movements substantially reduce the epidemic size associated with the dynamic model. Therefore, if network models are to be employed to investigate infectious diseases in the UK cattle herd, and used to make detailed quantitative predictions, then they should be based upon dynamic directed network representations of the available movement data.

Although these results all focus on the networks derived from the UK cattle movements, we believe that the general conclusions hold for a range of other network scenarios. For example, many human pathogens can be considered as spreading through a network of connections; while many family (or social) connections occur so frequently that they may be represented by a (weighted) static network, other connections are far more dynamic and are likely to have strong temporal correlations. As such, human contact patterns are most likely to be captured by a mixture of static and dynamic networks, with the dynamic links most often responsible for transmitting infection away from tightly clustered social cliques. Therefore, the general qualitative rules we have observed governing the differences between dynamic and static networks are likely to hold for human as well as livestock infections.

## Figures and Tables

**Figure 1:**
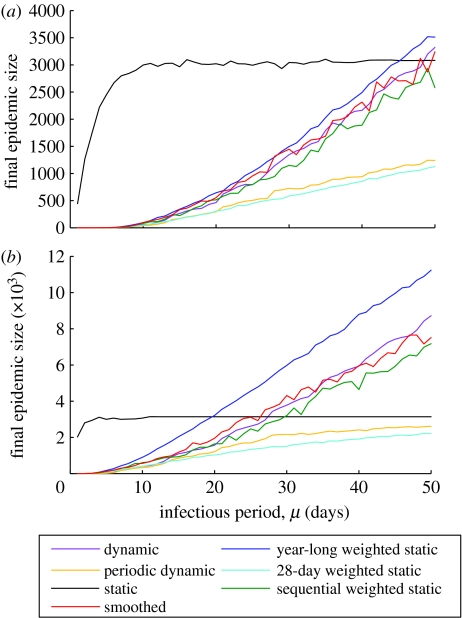
Infectious period versus final epidemic size for different representations of the UK cattle herd in 2004. Transmission probability, (*a*) *ν*=0.3, (*b*) *ν*=0.7.

**Figure 2:**
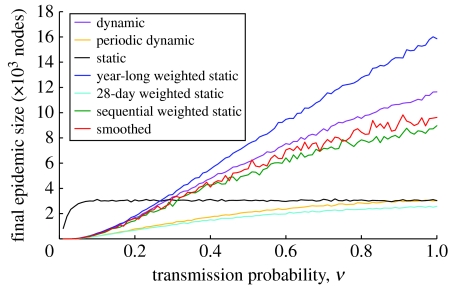
Final epidemic size against transmission probability for different representations of the UK cattle herd in 2004. Infectious period, *μ*=50 days.

**Figure 3:**
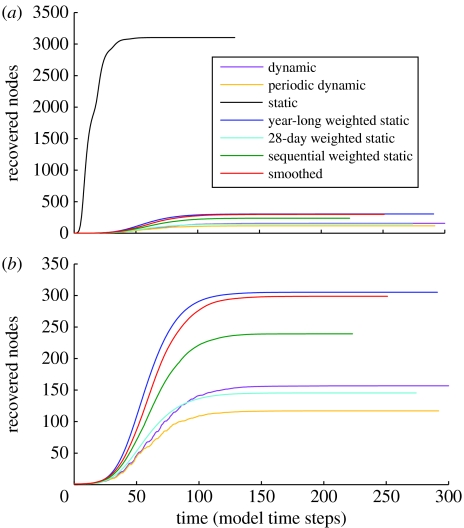
(*a*,*b*) Mean number of recovered nodes at each model time step for the different network representations; static network representation removed for clarity in (*b*). Transmission probability, *ν*=0.36; infectious period, *μ*=12.

**Figure 4:**
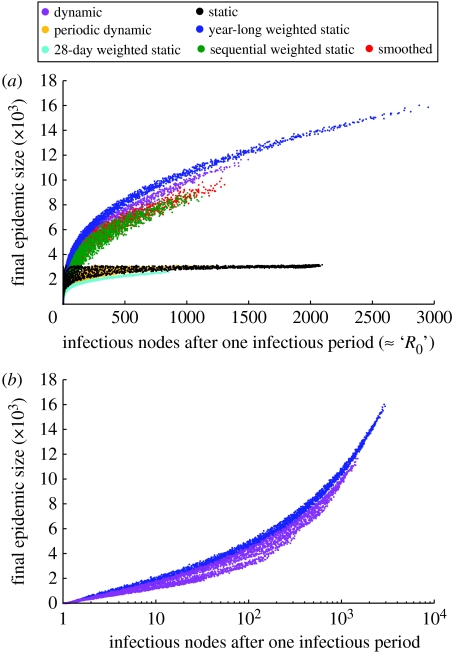
(*a*,*b*) Final epidemic size against number of infectious nodes after one infectious period: logarithmic *x*-axis and only two network representations shown, for clarity (*b*).

**Figure 5:**
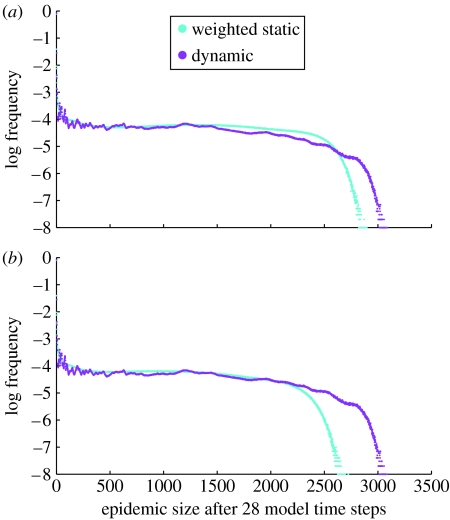
(*a*,*b*) Frequency distribution (from 100 000 000 runs) of final epidemic sizes from 28-day weighted static and dynamic network representations; simulations halted after 28 model time steps in each case. Transmission probability, *ν*=0.9; infectious period, *μ*=8 (*a*); transmission probability for 28-day weighted static representation adjusted to *ν*=0.8327 to give same mean final epidemic size as the dynamic network representation (*b*).
